# Gradients in compositions in the starchy endosperm of wheat have implications for milling and processing

**DOI:** 10.1016/j.tifs.2018.09.027

**Published:** 2018-12

**Authors:** Paola Tosi, Jibin He, Alison Lovegrove, Irene Gonzáles-Thuillier, Simon Penson, Peter R. Shewry

**Affiliations:** a School of Agriculture, Policy and Development, University of Reading, Whiteknights Campus, Early Gate, RG6 6AR, Reading, UK; b School of Science, Engineering and Design, Teesside University, TS1 3BA, UK; c Plant Science Department, Rothamsted Research, Harpenden, Herts, AL5 2JQ, UK; d Campden BRI, UK

**Keywords:** Wheat, Grain, Gradients, Starch, Protein, Milling

## Abstract

**Background:**

Wheat is the major food grain consumed in temperate countries. Most wheat is consumed after milling to produce white flour, which corresponds to the endosperm storage tissue of the grain. Because the starchy endosperm accounts for about 80% of the grain dry weight, the miller aims to achieve flour yields approaching this value.

**Scope and approach:**

Bioimaging can be combined with biochemical analysis of fractions produced by sequential pearling of whole grains to determine the distributions of components within the endosperm tissue.

**Key findings and conclusions:**

This reveals that endosperm is not homogeneous, but exhibits gradients in composition from the outer to the inner part. These include gradients in both amount and composition. For example, the content of gluten proteins decreases but the proportion of glutenin polymers increases from the outside to the centre of the tissue. However, the content of starch increases with changes in the granule size distribution, the proportions of amylose and amylopectin, and their thermal properties. Hence these parts of the endosperm differ in the functional properties for food processing. Gradients also exist in minor components which may affect health and processing, such as dietary fibre and lipids. The gradients in grain composition are reflected in differences in the compositions of the mill streams which are combined to give white flour (which may number over 20). These differences could therefore be exploited by millers and food processors to develop flours with compositions and properties for specific end uses.

## Highlights

• The wheat starchy endosperm comprises several cell types differing in composition.
• These differences include major functional components and healthy micronutrients.
• These gradients can be explored using microscopy, chemical imaging and pearling.
• These gradients are reflected in the compositions and properties of mill streams.
• Millers can exploit these differences to optimise flour composition.

## 1 Introduction

Cereal grains are the main source of food for humankind, with total global yields of about 2800 million tonnes (http://www.fao.org/faostat/en/#data), 90% of which is accounted by three major cereals: maize, rice and wheat. The cereal “grain” is actually a single seeded fruit, called a caryopsis, in which maternal pericarp and testa tissues surround the embryo and the endosperm, which represents the major storage tissue. The endosperm in turn comprises two distinct cell types: the aleurone cells, which have thick walls (and hence high fibre) and form the outermost layer, and the central starchy endosperm cells, which are rich in starch and gluten proteins (Barron, Surget, & Rouau, 2007). The outer grain layers and aleurone typically account for about 13–14% of the dry weight of the wheat grain, while the embryo and starchy endosperm account for 3% and 82–83%, respectively (Barron et al., 2007). Conventional milling separates the starchy endosperm cells form the other grain tissues, to give the white flour fraction which is widely used for making bread, other baked goods, pasta and noodles. A major aim of milling is therefore to maximise the recovery of white flour.

**Fig. 1:**
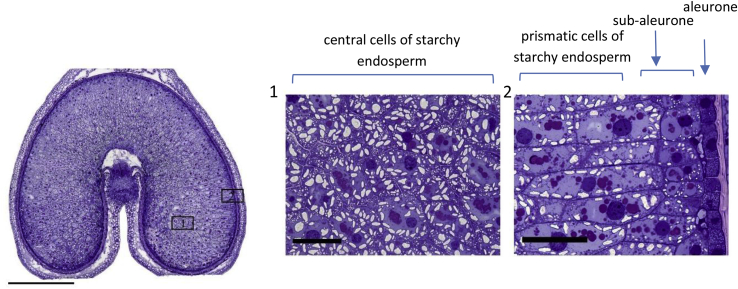
Cross section of a developing grain of durum wheat (cv Ofanto) at 20 days after anthesis, stained with toluidine blue to show the distribution of protein (taken from Tosi et al., 2009). The left hand image shows the whole grain with the areas in boxes 1 and 2 expanded in the central and right hand images, respectively. The bar in the cross-section represents 1 mm, the bars in panels 1 and 2 100 μm. Note the concentration of protein in the sub-aleurone cells in area 2. (For interpretation of the references to colour in this figure legend, the reader is referred to the Web version of this article.)

Although the starchy endosperm is usually treated as a single homogeneous tissue, it actually comprises several types of cells, which differ in their size and composition. This basic structure is illustrated in the micrographs of a developing grain of durum wheat in Fig. 1. A single layer of aleurone cells surrounds two to three layers of protein-rich sub-aleurone cells, with elongated prismatic cells radiating from these towards the centre of the grain (Fig. 1, area 2) and large central cells which are rich in starch being present in the centres of the cheeks (Fig. 1, area 1). Bradbury, MacMasters, and Cull (1956) reported approximate sizes of 60 μm diameter for the sub-aleurone cells, 128–200 μm x 40–60 μm for prismatic cells and 72-144 × 69–120 μm for the central cells. Differences in composition between these cell types have also been known for many years, with the sub-aleurone cells being richer in protein with fewer starch granules which are less regular in shape, compared with the other starchy endosperm cells (Bradbury et al., 1956; Kent, 1966; Kent & Evers, 1969). However, until recently these studies had been restricted to the gross distribution of proteins and starch.

More information on variation in composition within the starchy endosperm has come from studies using two approaches; high specificity antibodies combined with microscopy/bioimaging (Tosi et al., 2009; Tosi, Gritsch, He, & Shewry, 2011) and microspectroscopic imaging (notably FT-IR microspectroscopy (Toole et al., 2009, 2010)). These approaches have allowed more detailed resolution at the cell and tissue levels providing information on variation in the structures of components within the cells, as well as amounts and distributions. The sensitivity and resolution of immunomicroscopy is, however, still limited by lack of appropriate probes or masking caused by other components, and *in situ* imaging remains, at best, semi-quantitative. Therefore, it is necessary to combine such analyses with more traditional biochemical and chemical analyses of fractions.

**Fig. 2:**
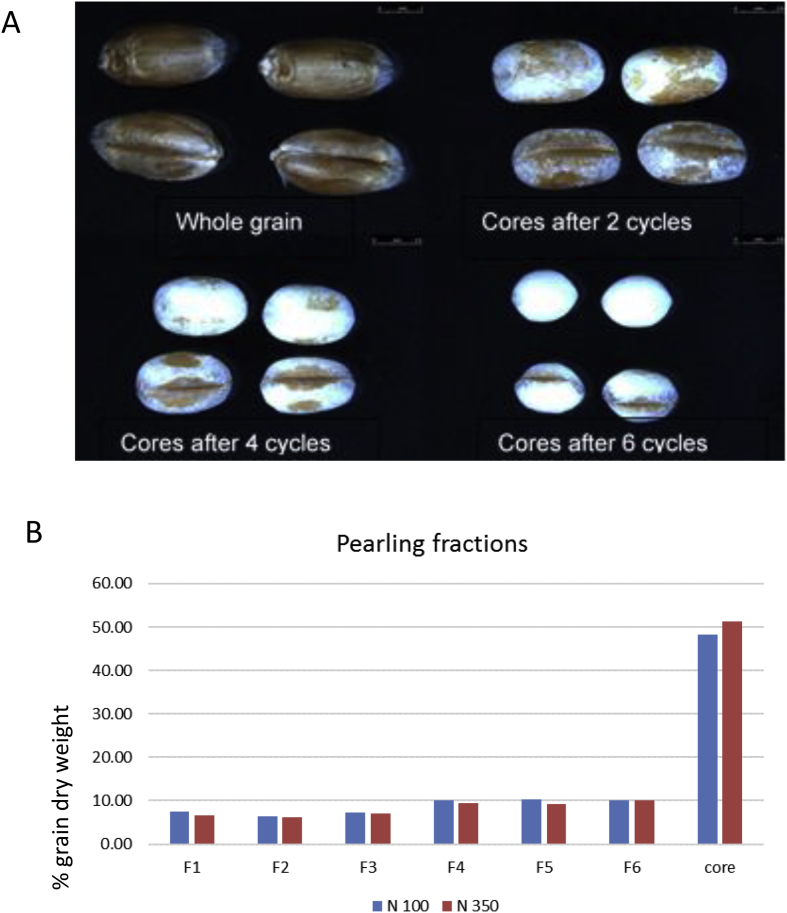
Pearling of grain of wheat cv Hereward. Part A shows the whole grain and the cores after a typical experiment of 6 pearling cycles. Part B shows the amounts of fractions removed during each pearling cycle (expressed as % total grain weight) from grain grown at 100 and 350 KgN/Ha. Taken from He et al. (2013).

Although it is possible to prepare small amounts of grain tissues by hand dissection (Barron et al., 2007) this approach cannot be taken to study endosperm gradients because of the hard and brittle nature of the tissue. The simplest approach is to progressively remove layers from the outside of the grain by friction (sometimes called peeling) or abrasion (pearling). This can be applied to substantial amounts of grain, but because of the elongated shape of the grain and the presence of a crease, the rate of removal is not uniform from the whole surface, being particularly high from the end of the grains (resulting in rounding). This is illustrated in Fig. 2, which shows the “cores” remaining after 2, 4 and 6 cycles of pearling, with between 6 and 10% of the grain weight being removed in each cycle. The remaining core can then be milled using a ball mill to give Fraction 7. Despite this uneven removal from different parts of the grain, in broad terms the fractions removed correspond initially to the pericarp and other outer layers, followed by the aleurone and then the sub-aleurone and outer parts of the starchy endosperm, with the core corresponding to the central starchy endosperm.

This article therefore brings data from pearling and other approaches to summarise our current knowledge of gradients in the mature wheat starchy endosperm, and discusses the implications of these for exploitation in innovative processing.

## 2 Gradients in grain composition

### 2.1 Protein

**Fig. 3:**
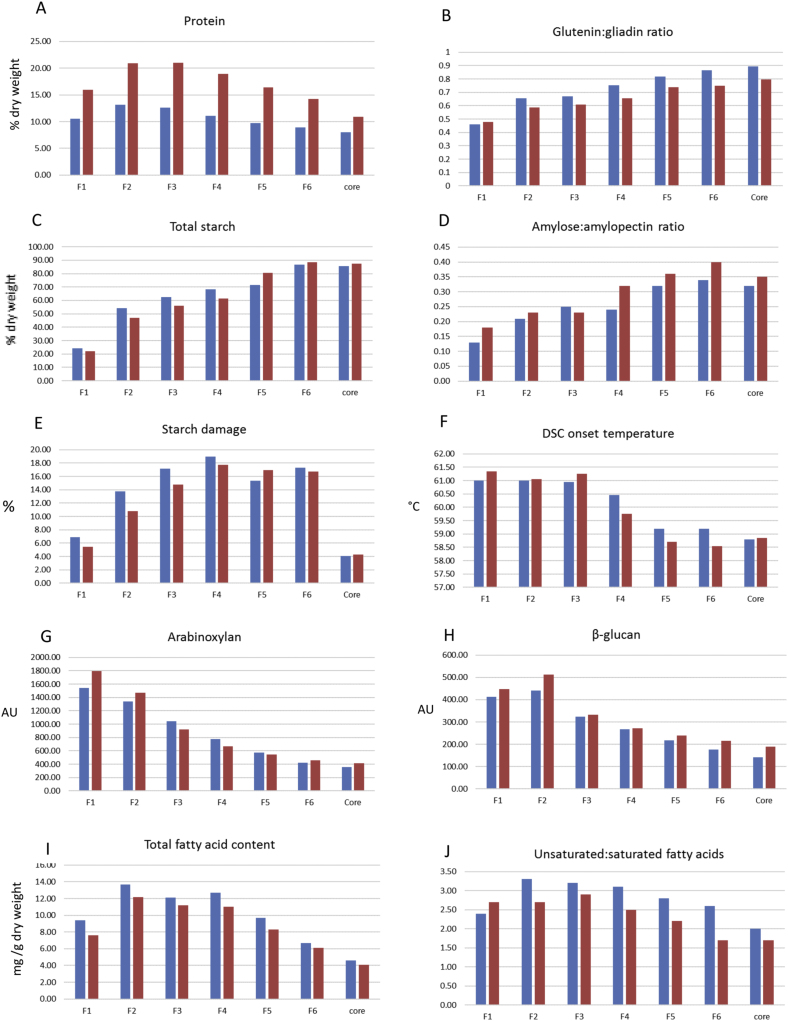
Distribution and properties of components in pearling fractions of wheat cv. Hereward, grown with 100 and 350m kgN/Ha. A, total protein; B, ratio of glutenin:gliadin proteins determined by SE-HPLC; C, total starch; D, ratio of amylose:amylopectin in starch; E, % starch damage; F, DSC onset temperature (^o^C); G, total arabinoxylan determined as arbitrary units; H, total β-glucan determined as arbitrary units; I, total lipids determined as fatty acids (% dry); J, ratio of unsaturated: saturated fatty acids. Statistic information for A and B was reported by He et al., 2013, differences among fractions was significant (p < 0.05) for C-D. Parts A and B are from He et al. (2013). Parts C-J used the same pearling fractions with Megazyme Assays for C, E, F, and methods described by Toole et al. (2010) (G, H) and Gonzáles-Thuillier et al. (2015) (I, J). Data in Part D was collected used a Pryis 1 DSC heating water-fractions mixture at 1:2 (g) ratio from 25 °C to 95 °C at a speed of 10 °C/min. To ensure comparability all analyses were carried out on the same three series of fractions from three replicate pearling experiments and means presented.

Fig. 3A shows a clear decrease in the concentration of protein (usually determined as nitrogen x 5.7) from the aleurone layer (which is enriched in pearling fraction 2) to the central starchy endosperm. This agrees with the early studies discussed above and with the study of Tosi et al. (2011) which combined microscopy with pearling. As would be expected, the protein contents of the fractions are also substantially higher in the grain grown at 350 kgN/Ha compared to grain grown at 100 kgN/Ha (Fig. 3A, p < 0.01 from analysis of variance test).

However, more detailed studies reveal more subtle gradients in protein composition. Thus, a combination of pearling and immunolabelling of tissue sections showed clear differences in the distributions of gluten proteins, with γ-gliadin and HMW subunits of glutenin being concentrated in the central starchy endosperm cells and α-gliadins, ω-gliadins and LMW subunits being concentrated in the outer layers (Tosi et al., 2011; He et al., 2013). This resulted in increases in the proportions of both total glutenin polymers and, in particular, high molecular mass glutenin polymers in the central part of the grain. The enrichment in glutenin polymers is illustrated in Fig. 3B, which shows the ratio of polymeric glutenins to monomeric gliadins determined by size exclusion HPLC. The proportion of high molecular mass glutenin polymers is strongly correlated with gluten strength and good bread making performance (reviewed by Shewry et al., 2003). Therefore, although the central starchy endosperm cells have a relatively low protein content, this protein would be expected to be of higher quality for bread making than the more abundant protein present in the outer starchy endosperm cells.

### 2.2 Starch

Starch is a mixture of two glucose polymers: amylose, which comprises single unbranched (1 → 4) α-linked chains of up to several thousand glucose units, and amylopectin which is highly branched (with (1 → 6) α-linkages as well as (1 → 4) α-linkages) and may comprise over 100,000 glucose units. The proportion of amylose in wheat starch generally ranges from about 18% to 35%. The proportions of amylose and amylopectin in starch have a significant impact on processing quality (as discussed below), with high amylopectin (waxy) starches being preferred for some food uses (Graybosch, 1998). By contrast, high amylose starches are attractive for developing healthy foods as they are more slowly digested in the human gastro-intestinal tract and become resistant on cooking, leading to reduced glycaemic index (Saris et al., 1998).

Starch is not present in the outer layers of the mature grain and hence the small proportions of starch present in pearling fractions 1 and 2 (Fig. 3C) can be assumed to be derived from the outer layers of the starchy endosperm (particularly from the ends of the grains). The content of starch increases from these fractions to the centre of the grain and represents about 80% of the weight in fraction 6 and the core (Fig. 3C). Mature wheat grain contains two distinct populations of starch granule, referred to as A-type and B-type, which differ in size and morphology (>10 μm and lenticular and <10μm and spherical, respectively). These populations also differ in polymer composition and structure (Shinde, Nelson, & Huber, 2003), with B-type granules containing lower proportions of amylose than larger granules (Duffus & Murdoch, 1979), and also differing in their swelling and gelatinization properties. Calorimetric studies have shown that isolated A-type granules have lower on-set gelatinization temperature and higher gelatinization enthalpy (ΔH*g*) than B-type granules. (Van Hung & Morita, 2005; Zeng, Li, Gao, & Ru, 2011). The two types of starch granules are unevenly distributed across the endosperm, with the sub-aleurone cells containing higher proportions of B-type granules compared to the central starchy endosperm cells (Tomlinson & Denyer, 2003). The lower proportions of amylose determined for the outer fractions of the grain (Fig. 3D) are therefore consistent with differences in the distributions of B-type and A–type granules in the different layers of endosperm cells.

The differences in starch granule size and composition in the pearling fractions would be expected to influence the functional properties of the flours. However, the gelatinization behaviour of starch differs between flour and isolated starch fractions, suggesting that it is influenced by the presence of other components in the flour. For example, gluten can shift the starch gelatinization range into higher temperatures (Eliasson, 1983; Eliasson, Gudmundsson, & Svensson, 1995) so that the onset temperature of gelatinization and the temperature at peak maximum of mill streams increase with increasing protein content although their *ΔH*_*g*_ (gelatinization enthalpy on a protein-free dry matter basis)- remains constant (Eliasson, Silverio, & Tjerneld, 1991). Furthermore, the gelatinization behaviour of starch is also affected by the degree of mechanical damage of starch during milling, with mill streams from conventional roller milling showing an inverse relationship between the amount of damaged starch and gelatinization enthalpy (ie enthalpy decreases with increasing of starch damage) (Eliasson et al., 1991; Jovanovich, Campana, Cardos, & Lupano, 2003).

The thermal properties of pearling fractions shown in Fig. 3F are in broad agreement with these published studies, with the onset temperature of gelatinization decreasing with increasing damaged starch, increasing amylose/amylopectin ratio and decreasing protein content. Although the forces applied by the pearling mill (used to prepare Fractions 1–6) and the ball mill (used to prepare Fraction 7 from the core) are substantially different from those occurring in conventional roller mills, with pearling resulting in much higher levels of starch damage (as shown in Fig. 3E), differences in thermal properties are nevertheless observed between fractions with similar levels of damage, indicating that they are relevant to commercial mill streams.

### 2.3 Non-starch polysaccharides (dietary fibre)

The non-starch polysaccharides present in cell walls are the major components of the dietary fibre fraction in wheat, accounting for about 11% of the grain dry weight (Andersson et al., 2013). However, there are well-documented differences in their content and composition between grain tissues. The outer layers of the mature wheat grain comprise about 45–50% cell wall material (Barron et al., 2007) which consists mainly of cellulose (30%), arabinoxylan (60%) and lignin (a phenolic polymer) (12%) (Stone & Morell, 2009). The thick cell walls of the aleurone cells account for about 35–40% of their dry weight and comprise mainly arabinoxylan (AX) (65%) and β-glucan (30%) (Stone & Morell, 2009), while the starchy endosperm cells have thin walls (about 2–3% dry weight) which also consist mainly of AX (70%) and β-glucan (20%) (Stone & Morell, 2009).

These differences in cell wall amount and composition are reflected in the pearling fractions, which show decreasing contents of AX and β-glucan towards the centre of the grain, with a small peak of β-glucan in pearling fraction 2, which may represent the glucan-rich walls of the aleurone cells. (Fig. 3 G and H). Saulnier et al. (2009) used a combination of microscale enzyme fingerprinting and *in situ* FT-IR microspectroscopic imaging to show differences in the proportions of AX and β-glucan between regions of the starchy endosperm, with a higher content of β-glucan in the outer layers close to the germ. Similarly, Dornez et al. (2011) used immunomicroscopy to show that β-glucan is concentrated in the walls of sub-aleurone cells. Saulnier et al. (2009) and Toole et al. (2010) also reported gradients in the fine structure of AX, with an increase in the proportion of xylose residues which are substituted with two arabinose residues from the outside to the inside of the endosperm. Analyses of developing grain using immunofluorescence microscopy indicate that the distributions of minor polysaccharides may also vary (Palmer et al., 2015), but these have not been studied in detail in mature grain and are unlikely to have significant effects on the overall composition and properties of commercial milling fractions.

### 2.4 Other components

Wheat grains contain many individual lipid components, which can be classified broadly into three types: storage triacylglycerols, polar lipids (phospholipids and glycolipids present in membranes) and free fatty acids (Gonzáles-Thuillier et al., 2015). All of these types of lipid exhibit wide diversity in structure, including differences in the polar head groups of phospholipids and glycolipids and in the acyl groups esterified to polar lipids and triacylglycerols. Determination of the total lipid total content of pearling fractions (as total fatty acids, including free fatty acids and fatty acids from acyl lipids) shows a high content in pearling fraction 2, which reflects the high content of triacylglycerols in the aleurone, with a decrease in concentration occurring from the outside to the centre of the starchy endosperm (Fig. 3I). Of particular interest is the proportions of unsaturated and saturated fatty acids, as polyunsaturated fatty acids are preferred for health but are more labile to oxidation during the storage of flours. Fig. 3J shows that the ratio of unsaturated to saturated fatty acids decreases from the aleurone to the centre of the grain. Small differences are also observed between the samples grown at low and high nitrogen fertilisation, with the latter showing a decreased proportion of saturated fatty acids in all fractions except fraction 1. More detailed studies of pearling fractions have shown that significant differences also exist in the distributions of the individual lipid components (Gonzáles-Thuillier et al. (2015), particularly the galactolipids monogalactosyldiglycerol (MGDG) and digalactosyldiglycerol (DGDG), which may affect the breadmaking performance of the flours (Pareyt, Finnie, Putseys, & Delcour, 2011).

A range of minor components are also present in the wheat grain and may influence the health benefits or processing properties. These include minerals, B vitamins (notably folates (B9) and vitamins B1, B2, B3 and B6) and phytochemicals (notably phenolics and terpenoids). These components are all known to be concentrated in the bran, (see, for example, Adom, Sorrells, and Liu (2005)), but less is known about their distribution within the starchy endosperm. Minerals are particularly problematic to map because they are present at very low levels in the starchy endosperm and at very high levels in the aleurone layer (mainly as phytates). However, a pearling study which included the fractions in Fig. 2 (Xue et al., 2014) showed a progressive decrease in total iron and zinc from the outer layers to the central starchy endosperm, but an increase in the proportion of low molecular weight soluble forms. Since the major forms of minerals in the aleurone are insoluble phytates (Schlemmer, Frølich, Prieto, & Grases, 2009), this increase in soluble iron may represent an increasing proportion of minerals derived from the central endosperm cells: this is important as minerals bound to phytate have low bioavailability, while the soluble forms present in white flours are highly bioavailable (Eagling, Wawer, Shewry, Zhao, & Fairweather-Tait, 2014). Although methods are available to map metals in biological tissues *in situ* (reviewed by Heard, Feeney, Allen, & Shewry, 2002), the levels of iron and other minerals in the starchy endosperm are generally too low to allow analysis at the tissue level (De Brier et al., 2016).

## 3 Significance of gradients in grain composition for food processing

Because the wheat grain is elongated and presents a crease, pearling fractions do not correspond to pure grain tissues Nevertheless, combining the analysis of pearling fractions with other approaches (microscopy, imaging and hand dissection of tissues) demonstrates that gradients in composition exist within the starchy endosperm, the tissue which gives white flour on milling (summarised in Table 1). These include gradients in the proportions of major (proteins, starch) and minor (fibre, lipids) components and differences in the fine structures of these. Furthermore, effects may be modulated by crop nutrition. Consequently, pearling fractions differ significantly in their processing properties (as recently demonstrated by Zhong et al., 2018) and in their contents of components that contribute to diet and health.

**Table 1:** Summary of gradients in composition within the starchy endosperm of wheat grain.

Component	Gradient from *outer* to *inner* starchy endosperm	Implications for processing and health
**Protein**		
Total protein (% dry wt)	Decrease	High protein content and high proportion of gluten polymers have positive effects on flour quality for breadmaking
Gluten proteins (% total protein)	Increase	
Proportions of glutenins and large glutenin polymers (% gluten proteins)	Increase	
**Starch**		
Total starch (% dry wt)	Increase	Starch content, amylose:amylopectin ratio and gelatinization temperature all affect processing properties. High amylose starch has lower glycaemic index
A-type granules (% total granules)	Increase	
% amylose	Increase	
DSC onset temperature	Decrease	
**Dietary fibre**		
Total arabinoxylan (% dry wt)	Decrease	High dietary fibre has established health benefits.
Arabinoxylose substitution (% disubstituted xylose residues))	Increase	
Total β-glucan (% dry wt)	Decrease	
**Lipids**		
Total lipids (% dry wt)	Decrease	Unsaturated fatty acids preferred for health but can lead to rancidity during storage. Lipid composition affects breadmaking quality
Unsaturated fatty acids (% total fatty acids)	Decrease	

Although pearling is often used commercially to remove the outer layers of the grain before milling, it is not a practical alternative to roller milling for the commercial production of white flour. However, analyses of white flour fractions from roller milling show that they also differ in composition, to a similar extent to the differences observed between the more central pearling fractions (Gonzáles-Thuillier et al., 2015; Nyström, Paasonen, Lampi, & Piironen, 2007; Prabhasankar, Sudha, & Haridas R, 2000; Ramseyer, Bettge, & Morris, 2011). Although differences in the compositions of mill streams are often assumed to relate to differences in the extent of contamination with bran, we consider that they also relate to the origin of the flour fractions from different parts of the grain. Hence, the purest first break and reduction fractions are probably derived from the central starchy endosperm cells and the later breaks and reductions to more peripheral regions. These differences could therefore be exploited by millers and food processors, to develop flours with compositions and properties for specific end uses.

For example, flour fractions enriched in central endosperm cells which have a higher content of high molecular mass glutenin polymers and a higher ratio of glutenin subunits to gliadins (He et al., 2013) would be expected to provide doughs of higher elasticity but lower viscosity than doughs produced with higher extraction flours. These characteristics are particularly sought after in breadmaking systems requiring flours of high strength, notably the Chorleywood Breadmaking Process which is widely used in the UK and a number of other countries. By contrast, flour streams enriched in the outer layer of the endosperm would have lower contents of starch and a lower ratio of glutenin subunits to gliadins and therefore expected to produce doughs of higher extensibility which may be required for bakery products other than leavened breads, notably biscuits. The same fractions may also have sufficient extensibility and tenacity to be incorporated into pasta making dough for the preparation of fresh or dry “special pasta”. Consequently, although high extraction flours from a certain wheat crop may not meet protein quality standards for a specific product, some of the flour fractions could be suitable.

Differences in amylose:amylopectin ratio between flour millstreams, in combination with differences in protein composition, may also be exploited to improve “processability”, by increasing texture resilience of wheat-based foods requiring frozen/chilled technology. For example, chilled doughs, bake–at-home breads and frozen cookie doughs.

Finally, specific wheat millstreams, including middling fractions, could also be selectively recombined to obtain flours with specific health and nutritional benefits. For example, enrichment in specific type of dietary fibre (for example β-glucan in sub-aleurone fractions), phytochemicals, minerals and high quality proteins derived from partial incorporation of the aleurone layer. Such novel flour formulations could be exploited to improve the nutritional/health credentials of wheat-based foods and meet the requirements of more health-conscious consumers.

## Funding

Rothamsted Research receives grant-aided support from the Biotechnology and Biological Sciences Research Council of the UK and the work at Rothamsted forms part of the Designing Future Wheat strategic programme [BB/P016855/1). The work was also supported by a BBSRC Industrial CASE studentship to JH, and by BBSRC grant BB/J019526/1.

## Declarations of interest

None.
